# Skeletal muscle cutoff values for sarcopenia diagnosis using T10 to L5 measurements in a healthy US population

**DOI:** 10.1038/s41598-018-29825-5

**Published:** 2018-07-27

**Authors:** Brian A. Derstine, Sven A. Holcombe, Brian E. Ross, Nicholas C. Wang, Grace L. Su, Stewart C. Wang

**Affiliations:** 1 Morphomic Analysis Group, University of Michigan, Ann Arbor, MI USA; 2 Department of Internal Medicine, University of Michigan, Ann Arbor, MI USA; 3 Department of Medicine, VA Ann Arbor Healthcare System, Ann Arbor, MI USA; 4 Department of Surgery, University of Michigan, Ann Arbor, MI USA

## Abstract

Measurements of skeletal muscle cross-sectional area, index, and radiation attenuation utilizing clinical computed tomography (CT) scans are used in assessments of sarcopenia, the loss of skeletal muscle mass and function associated with aging. To classify individuals as sarcopenic, sex-specific cutoffs for ‘low’ values are used. Conventionally, cutoffs for skeletal muscle measurements at the level of the third lumbar (L3) vertebra are used, however L3 is not included in several clinical CT protocols. Non-contrast-enhanced CT scans from healthy kidney donor candidates (age 18–40) at Michigan Medicine were utilized. Skeletal muscle area (SMA), index (SMI), and mean attenuation (SMRA) were measured at each vertebral level between the tenth thoracic (T10) and the fifth lumbar (L5) vertebra. Sex-specific means, standard deviations (s.d.), and sarcopenia cutoffs (mean-2 s.d.) at each vertebral level were computed. Associations between vertebral levels were assessed using Pearson correlations and Tukey’s difference test. Classification agreement between different vertebral level cutoffs was assessed using overall accuracy, specificity, and sensitivity. SMA, SMI, and SMRA L3 cutoffs for sarcopenia were 92.2 cm^2^, 34.4 cm^2^/m^2^, and 34.3 HU in females, and 144.3 cm^2^, 45.4 cm^2^/m^2^, and 38.5 HU in males, consistent with previously reported cutoffs. Correlations between all level pairs were statistically significant and high, ranging from 0.65 to 0.95 (SMA), 0.64 to 0.95 (SMI), and 0.63 to 0.95 (SMRA). SMA peaks at L3, supporting its use as the primary site for CT sarcopenia measurements. However, when L3 is not available alternative levels (in order of preference) are L2, L4, L5, L1, T12, T11, and T10. Healthy reference values reported here enable sarcopenia assessment and sex-specific standardization of SMA, SMI, and SMRA in clinical populations, including those whose CT protocols do not include L3.

## Introduction

Sarcopenia is an important aspect of malnutrition diagnosis^1^ and a risk factor for poor clinical outcomes^2–10^. Consensus recommendations for its diagnosis define sarcopenia as both low muscle function (performance or strength) and low muscle mass, using sex-specific cutoffs for ‘low’ values^11–15^. Cutoffs set at two standard deviations below the mean of a healthy, young adult population were recommended by the European Working Group on Sarcopenia in Older People (EWGSOP)^11^. In addition to gait speed for performance and handgrip testing for strength, gold standards for measurement include computed tomography (CT) for muscle mass. For research purposes such measurements are often collected retrospectively and opportunistically; extracting measurements from CT scans that were obtained during the normal course of clinical care adds no additional risk and is convenient. CT measurements of lumbar skeletal muscle area (SMA) have been shown to be strongly correlated with whole body muscle mass in healthy adults^16–19^, while mean skeletal muscle radiation attenuation (SMRA), a measure of muscle fat content, has been shown to be associated with physical function^18,20–22^. SMA or height-adjusted SMA (SMI) at the third lumbar vertebra (L3) is prevalent in sarcopenia assessments of low muscle mass related to poor clinical outcomes^2–10^. In the absence of functional testing data, SMRA could be used as a surrogate; SMA or SMI, combined with SMRA, would enable a complete sarcopenia assessment from CT alone, given the appropriate cutoff values from a healthy reference population.

Reference values from a young, healthy population provide a necessary basis for comparisons between different clinical populations, and any sarcopenia cutoffs derived therefrom. Such cutoffs for low L3 skeletal muscle measurements have been previously reported for US and European cohorts^10,23^ and for L3 psoas muscle in an Asian cohort^9^.

Because clinical CT protocols do not always include L3, we previously reported T12 values as an alternative for chest-only CT protocols. However, there is no practical reason why skeletal muscle evaluation should be limited to these two levels. Indeed, Shen *et al*.^17^ reported high correlations around L4-L5 suggesting that these would be suitable alternatives to L3. Our primary aim is to define means, standard deviations, and sarcopenia cutoffs in males and females at all vertebral levels from T10 to L5, enabling a broader range of CT protocols to be used in assessments of sarcopenia and facilitating comparisons of clinical populations to a healthy reference.

## Results

### Population Summary

The majority of subjects (494, 67%) eventually donated a kidney, the remainder (241, 33%) had no record of donation (Table 1). There were no significant sex differences in mean age (31.2/30.9 yr, p > 0.53), donor proportion (69.2/64.7%, p > 0.20), mean BMI (26.9/27.6 kg/m^2^, p > 0.069), or tube current (196.5/199.9 mA, p > 0.25). Males had significantly (p < 0.001) greater height (164.2/179.1 cm) and weight (72.7/88.7 kg).

**Table 1:** Cohort demographics and CT parameters split by sex.

	Female		Male		*p*
	n	mean ± s.d.	N	mean ± s.d.	
Age (yr)	415	31.2 ± 6.1	320	30.9 ± 6.1	0.531
Height (cm)	415	164.2 ± 6.9	320	179.1 ± 7.1	<0.001
Weight (kg)	402	72.7 ± 15.9	308	88.7 ± 16.4	<0.001
BMI (kg/m^2^)	402	26.9 ± 15.9	308	27.6 ± 16.4	0.069
*Underweight* < 18.5	6	1.4%	1	0.3%	
*Normal* (18.5–25)	171	41.2%	79	24.7%	
*Overweight* (25–30)	102	24.6%	153	47.8%	
*Obese class I* (30–35)	87	21.0%	49	15.3%	
*Obese class II* (35–40)	26	6.3%	24	7.5%	
*Obese class III* >= 40	10	2.4%	2	0.6%	
*NA*	13	3.1%	12	3.8%	
Donor					0.206
*Yes*	287	69.2%	207	64.7%	
*No*	128	30.8%	113	35.3%	
Tube Current (mA)	415	196.5 ± 43.1	320	199.9 ± 37.9	0.253

Donor proportion p-value from Chi-squared test, all others from t-test.

### Skeletal Muscle Measurements

**Figure 1:**
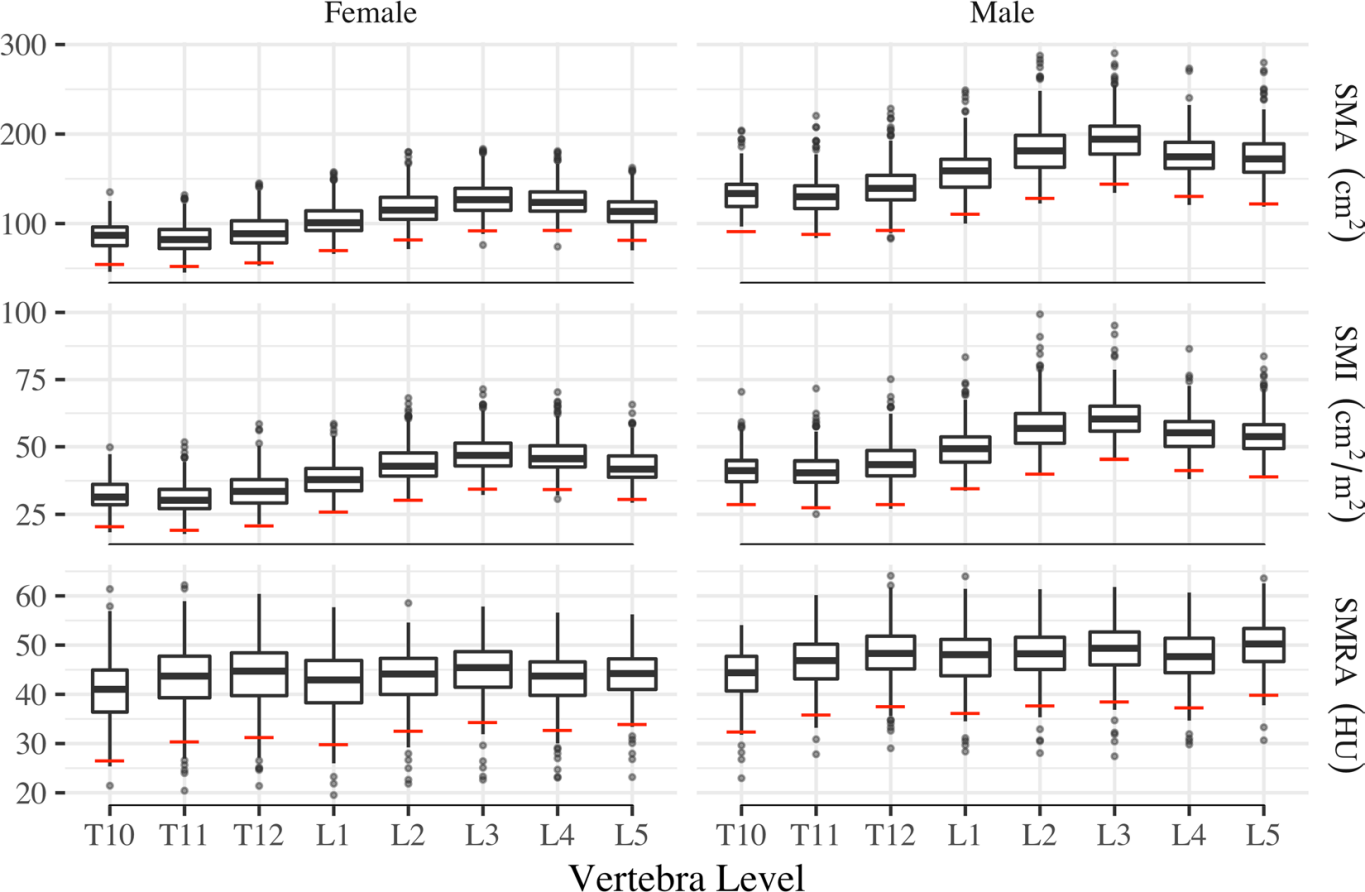
Box plots of healthy reference population SMA, SMI, and SMRA values by sex and vertebra. EWGSOP sarcopenia cutoffs (mean − 2 s.d.) shown as horizontal red lines.

Sex-specific mean, standard deviation (s.d.), and cutoff values for SMA, SMI, and SMRA at all vertebra levels are reported in Table 2. Male SMA, SMI, and SMRA were significantly (p < 0.001) greater than corresponding female means at all levels. Peak and trough mean SMA and SMI values were observed at L3 and T11, respectively, in both females and males (Fig. 1). Mean SMA ranged from 83.5–128 cm^2^ in females, and 131.7–195.2 cm^2^ in males.

**Table 2:** Sex-specific healthy reference values for T10-L5 SMA, SMI, and SMRA.

	VB	Female			Male			p
		n	mean ± s.d.	cutoff	N	mean ± s.d.	cutoff	
SMA (cm^2^)	T10	156	86.6 ± 16.0	54.6	122	135.3 ± 22.0	91.4	<0.001
	T11	336	83.5 ± 15.5	52.4	241	131.7 ± 21.8	88.1	<0.001
	T12	401	91.3 ± 17.6	56.1	299	141.2 ± 24.4	92.3	<0.001
	L1	409	103.4 ± 16.7	70.1	315	159.2 ± 24.4	110.4	<0.001
	L2	411	117.7 ± 17.9	81.9	315	183.9 ± 27.8	128.2	<0.001
	L3	410	128.0 ± 17.9	92.2	317	195.2 ± 25.4	144.3	<0.001
	L4	399	125.5 ± 16.6	92.4	305	177.1 ± 23.2	130.7	<0.001
	L5	295	114.9 ± 16.7	81.5	211	176.0 ± 27.0	122.0	<0.001
SMI (cm^2^/m^2^)	T10	156	32.3 ± 5.9	20.4	122	42.2 ± 6.7	28.8	<0.001
	T11	336	31.0 ± 5.9	19.2	241	41.1 ± 6.8	27.6	<0.001
	T12	401	34.0 ± 6.6	20.8	299	44.1 ± 7.7	28.8	<0.001
	L1	409	38.4 ± 6.2	25.9	315	49.7 ± 7.6	34.6	<0.001
	L2	411	43.7 ± 6.7	30.4	315	57.4 ± 8.7	40.1	<0.001
	L3	410	47.5 ± 6.6	34.4	317	60.9 ± 7.8	45.4	<0.001
	L4	399	46.7 ± 6.2	34.2	305	55.3 ± 7.0	41.3	<0.001
	L5	295	42.8 ± 6.1	30.6	211	54.7 ± 7.9	39.0	<0.001
SMRA (HU)	T10	156	40.4 ± 6.9	26.5	122	43.8 ± 5.7	32.4	<0.001
	T11	336	43.4 ± 6.5	30.4	241	46.5 ± 5.3	35.8	<0.001
	T12	401	44.0 ± 6.4	31.3	299	48.2 ± 5.3	37.5	<0.001
	L1	409	42.6 ± 6.4	29.8	315	47.5 ± 5.7	36.2	<0.001
	L2	411	43.4 ± 5.5	32.5	315	48.1 ± 5.2	37.7	<0.001
	L3	410	44.9 ± 5.3	34.3	317	49.0 ± 5.3	38.5	<0.001
	L4	399	43.1 ± 5.2	32.7	305	47.6 ± 5.2	37.3	<0.001
	L5	295	43.9 ± 5.0	33.9	211	49.9 ± 5.0	39.8	<0.001

**Figure 2:**
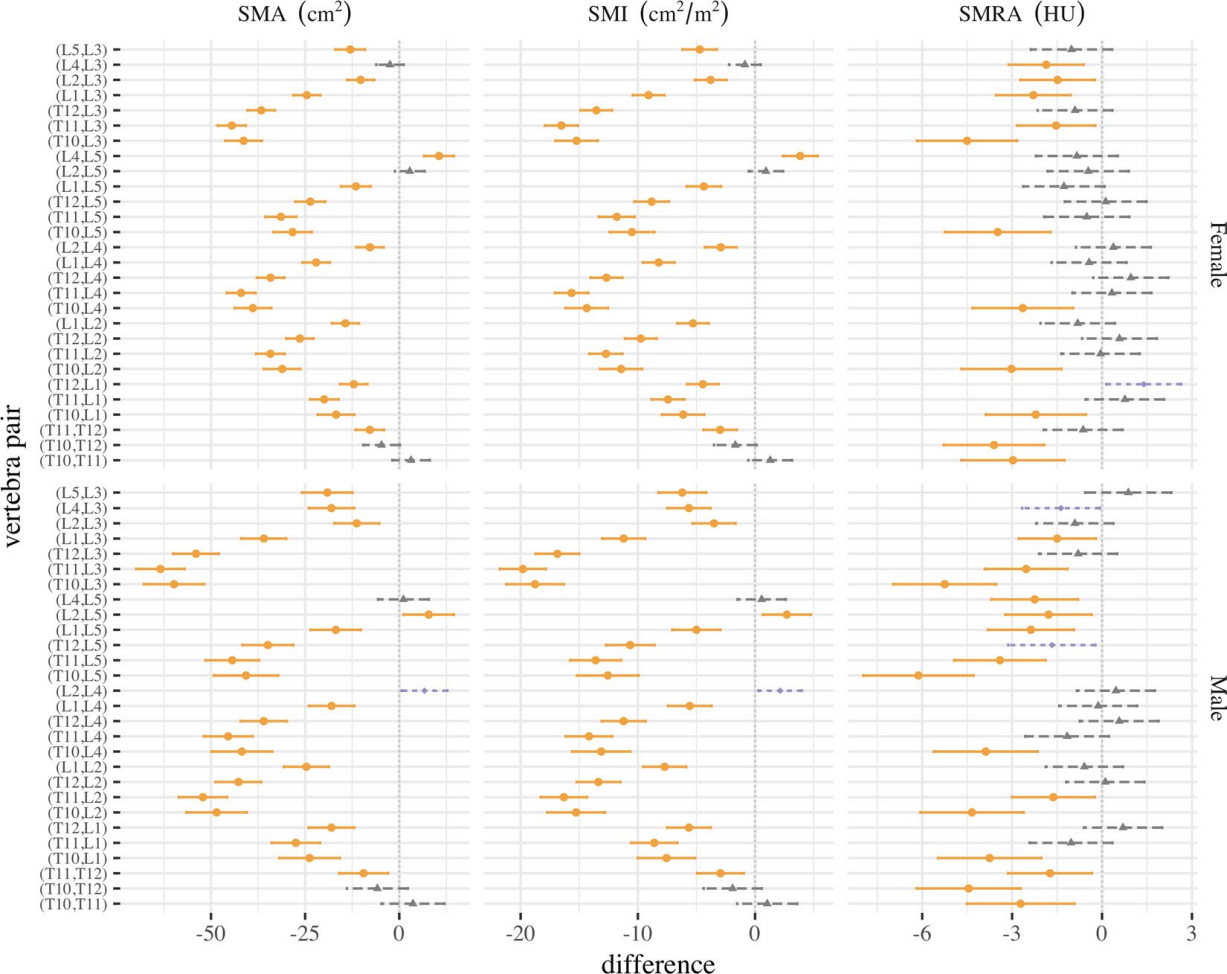
Tukey mean difference and 95% confidence interval for SMA, SMI, and SMRA at each vertebral level pair, by sex. Differences with p < 0.01 (orange circles), p < 0.05 (purple diamonds), and p > 0.05 (grey triangles) are shown.

All SMA or SMI mean differences were significantly different (p < 0.01) *except* for six vertebral pairs: (T10, T11) and (T10, T12) in both sexes, (L2, L5) and (L3, L4) in females, and (L2, L4) and (L4, L5) in males (Supplemental Tables S1 and S2). SMRA mean differences were significantly different for seventeen pairs: (T11, L3), (L1, L3), and all seven T10 vertebral pairs in both sexes, (L2, L3) and (L4, L3) in females, and (T11, T12), (T11, L2), (T11, L5), (L1, L5), (L2, L5), and (L4, L5) in males (Supplemental Table S3). SMRA in all other pairs was not significantly different (p > 0.01) (Fig. 2). The smallest differences in means compared to L3 SMA and SMI were L2, L4, and L5, while for SMRA there was not a clear pattern.

Pearson correlations between all levels were statistically significant (p < 0.001) and high, ranging from 0.65 to 0.95 (SMA), 0.64 to 0.95 (SMI), and 0.63 to 0.95 (SMRA) (Supplemental Tables S4–S6). Correlations were highest between adjacent levels and lowest between the farthest apart levels (e.g., T10, L5). The levels that were most highly correlated with L3 SMA were L2 (females: 0.94, males: 0.92), L1 (0.91/0.87), L4 (0.88/0.88), and L5 (0.85/0.84). The same levels were most highly correlated with L3 SMI. L3 SMRA was most highly correlated with L2 (0.94/0.95), L4 (0.92/0.93), L5 (0.87/0.93), and L1 (0.88/0.91).

Overall accuracy of all vertebral level cutoffs (compared against L3) was high, ranging from 95% to 100%. Specificity was also high, ranging from 97% to 100%. However, sensitivity varied widely, ranging from 0% to 100%, partly due to the low number of sarcopenic individuals in the dataset (Table 3).

**Table 3:** Accuracy of binary cutoffs by vertebra level and sex using the L3 classifier as reference. (L3 Low = the number of reference cases classified as “low”, e.g., L3 value below the L3 cutoff. Acc. = Overall accuracy. Sens. = sensitivity. Spec. = specificity).

	VB	Female					Male				
		N	L3 Low	Acc.	Sens.	Spec.	N	L3 Low	Acc.	Sens.	Spec.
SMA	T10	156	2	98.7	50.0	99.4	122	0	100.0	*NA*	100.0
	T11	335	4	99.4	50.0	100.0	241	1	99.2	100.0	99.2
	T12	400	6	98.8	33.3	99.7	299	3	99.3	66.7	99.7
	L1	408	6	99.3	66.7	99.8	315	4	99.4	75.0	99.7
	L2	410	6	99.8	83.3	100.0	315	4	99.7	75.0	100.0
	L4	398	5	98.0	20.0	99.0	305	4	98.7	25.0	99.7
	L5	294	5	98.6	20.0	100.0	211	3	100.0	100.0	100.0
SMI	T10	156	2	99.4	100.0	99.4	122	0	100.0	*NA*	100.0
	T11	335	3	99.1	33.3	99.7	241	1	98.8	0.0	99.2
	T12	400	3	99.2	0.0	100.0	299	1	99.0	0.0	99.3
	L1	408	3	99.3	33.3	99.8	315	1	98.7	0.0	99.0
	L2	410	3	99.5	33.3	100.0	315	1	99.7	0.0	100.0
	L4	398	3	99.5	66.7	99.7	305	1	99.0	0.0	99.3
	L5	294	3	99.3	66.7	99.7	211	0	99.5	*NA*	99.5
SMRA	T10	156	9	95.5	33.3	99.3	122	6	97.5	66.7	99.1
	T11	335	12	98.5	75.0	99.4	241	8	95.0	37.5	97.0
	T12	400	12	98.5	83.3	99.0	299	8	98.7	75.0	99.3
	L1	408	12	99.0	75.0	99.7	315	8	97.8	62.5	98.7
	L2	410	12	99.0	75.0	99.7	315	8	99.4	87.5	99.7
	L4	398	12	98.2	75.0	99.0	305	9	98.0	55.6	99.3
	L5	294	9	98.0	66.7	98.9	211	6	98.6	66.7	99.5

## Discussion

Torso CT protocols appropriate for measuring skeletal muscle generally fall into three categories: chest (T1-to-L1), abdomen (T10-to-L4), and pelvis (L4-to-L5). Measurements of skeletal muscle for the identification of sarcopenia are typically taken at the level of the third lumbar vertebra. Our results demonstrate that L3 measurements are significantly different from other vertebral levels. Given these limitations, L3 sarcopenia cutoffs only apply to patients who receive abdominal CT imaging, excluding those who receive imaging of the chest only (e.g., lung cancer screening) and of the pelvis without abdomen (e.g., pelvis or hip fracture). The values reported here expand sarcopenia evaluations to these chest and pelvis protocols to enable wider clinical applications. We had previously reported L3 means/cutoffs in females as 126.8/91.2 (SMA) and 47.0/34.0 (SMI), and in males as 190.9/141.7 (SMA) and 59.7/44.6 (SMI); and T12 means/cutoffs in females as 90.8/55.9 (SMA) and 33.7/20.6 (SMI), and in males as 138.6/91.5 (SMA) and 43.3/28.7 (SMI)^23^. The updated mean values presented here were neither statistically nor clinically significantly different from the previous values, and the cutoffs were within 2% of previous. Werf *et al*. (p.292)^10^ reported 5^*th*^ percentile cutoff values for L3 SMA in a healthy European Caucasian population (age 20–40) as ‘44.7 cm^2^/m^2^ in men and 33.0 cm^2^/m^2^ in women’, within 2% (males) and 4% (females) of our reported values. Cutoffs reported for age 20–82 are not comparable due to the inclusion of those over age 40.

Minor variations in reference cutoff values, and the low sensitivity of different vertebral level cutoffs found here, demonstrate a potential difficulty of treating sarcopenia as a binary classification rather than a continuum. Single number cutoffs for defining sarcopenia, while convenient for determining prevalence of sarcopenia in a particular cohort, may not adequately capture individual risk, especially for patients whose measurements are near cutoff values. Muscle mass and function decline with age, which means older, more sarcopenic cohorts may have little practical use for single number cutoffs in statistical models^24^. However, the sex-specific young adult means and standard deviations we have presented can be easily used to standardize any individual measurement (e.g., *SMA*_*tscore*_ = (*SMA* − *mean*)/*s*.*d*.). Standardization of predictor variables is a standard practice in regression modeling^25^, and results in muscle measures that are akin to the bone mineral density ‘T-score’^26^. Standardizing SMA, SMI, or SMRA in this manner has the added benefit of normalizing male and female values to the same scale, controlling for sex differences.

This study has important limitations. Our cohort may not be nationally or globally representative and is not specific to a particular race or ethnicity. Our population-based cutoffs have not been tested against clinical outcomes. We used non-contrast-enhanced CT scans; previous research has shown that IV contrast has a clinically insignificant effect on SMA^23,27,28^, but a larger, more significant effect on SMRA^27,28^. Care should be exercised when applying these reference values in IV contrast-enhanced CT images and those using different kVp, convolution kernel, and/or slice thicknesses.

Clinical CT scans obtained in the normal course of patient care can potentially be used for sarcopenia evaluations. However, scans may not include vertebral levels for which sarcopenia cutoffs have been defined (e.g., L3). We updated previously published healthy population reference values for SMA and SMI at T12 and L3, included SMRA, a measure of muscle function, and reported on a wider range of vertebral levels to enable sarcopenia evaluations on a wider range of CT imaging protocols. We calculated female and male mean and standard deviation for each level from T10 to L5, and we followed EWGSOP consensus recommendations to define sex-specific sarcopenia cutoffs as two standard deviations below the mean of a healthy, young adult cohort. Further research is needed to determine the relationship between cutoffs at various vertebral levels and clinical outcomes. While L3 remains the ideal location for skeletal muscle measurement, results indicate that (in order) L2, L4, L5, L1, T12, T11, and T10 are the preferred alternatives when L3 is unavailable.

## Methods

### Study Cohort

We retrospectively studied persons who underwent CT scans at the University of Michigan as part of evaluation for kidney donation between 1999 and 2011. We have previously studied a subset of these kidney donor candidates as a healthy reference population^23^.

Patient age, sex, height, and weight were obtained from their medical record proximal to the date of evaluation for kidney donation, and the month and year of the evaluation appointment was recorded^29^. Candidates were included if they had a CT scan performed as part of evaluation for kidney donation, were deemed healthy enough to donate, had age, sex, and height recorded in their electronic medical record, had non-contrast-enhanced series available, and had a fascia boundary that was fully visible in the display field of view.

Though inconsequential to the current analysis, weight was not retrospectively available for *n* = 25 (3.4%) subjects. Body mass index (BMI) was computed and categorized into groups according the World Health Organization International Classification standard^30^. Race, unavailable for 52% of the cohort, was not specifically accounted for in the analysis.

CT imaging was extracted for 1,482 total donor candidates between the ages of 18 and 70. The analysis cohort included *n* = 735 ‘young adult’ candidates between the age of 18 and 40^31–35^ scanned using the GE ‘Standard’ reconstruction algorithm at 120 kVp and 5 mm slice thickness in a Discovery or LightSpeed scanner. Tube current was automatically modulated in proportion to body mass.

### CT Image Processing

**Figure 3:**
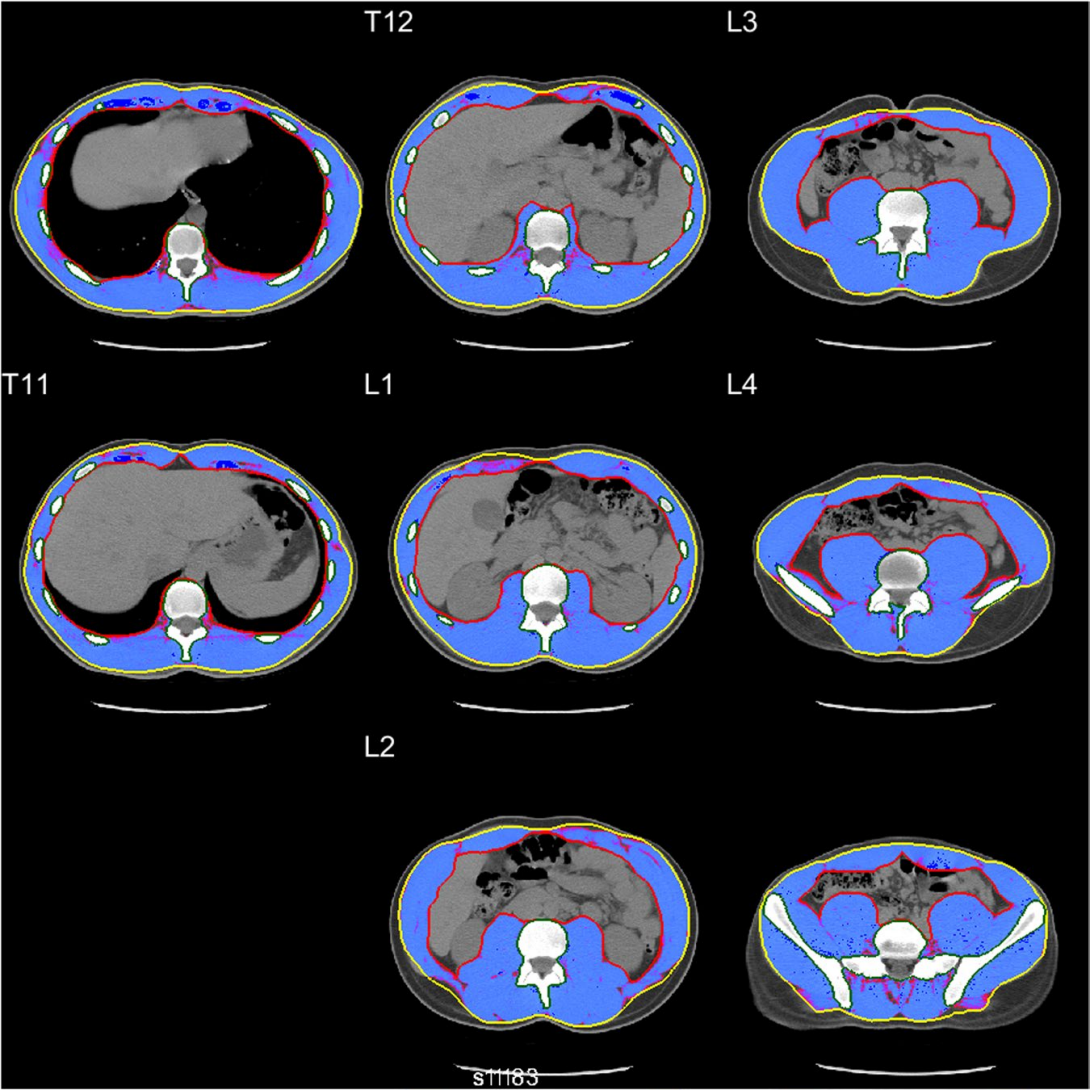
Example of healthy 20 y/o male T10-L5 axial CT slices showing SMA (blue-shaded area) between outer abdominal fascia (yellow line) and inner muscle wall (red line).

After being transferred into a spatial database, CT images were processed using Analytic Morphomics, a semi-automated image analysis method that has been previously described^23,36^. A combination of automated and user-guided algorithms written in Matlab (The Mathworks Inc, Natick, MA) identified the vertebral bodies to serve as an anatomical coordinate reference system. Next, the outer abdominal fascia and inner muscle wall were identified at all available vertebral levels to create enclosed regions of interest, which were confirmed by multiple trained researchers (Fig. 3).

Sample size at each vertebral level varied due to differences in anatomy included in each scan. Measurements at T8 and T9 were excluded due to statistically significant differences in mean weight compared to those at T10 through L5. For T10-L5, there there were no significant differences in mean age, weight, or height within the male and female cohorts.

SMA was measured at the axial slice nearest the inferior aspect of each vertebral body as the area of pixels within −29 to +150 Hounsfield Units (HU) as previously validated^21,23,27^. Skeletal muscle index (SMI)–a heuristic that normalizes muscle area for height–was computed as SMA divided by height-squared^37^. Skeletal muscle radiation attenuation (SMRA) was computed as the mean Hounsfield Unit (HU) value of all pixels included in SMA^27,38,39^.

### Statistical Methods

Male and female demographics, CT parameters, and skeletal muscle measurements are shown separately as mean +/− s.d. for continuous variables and proportion for categorical variables. Means were compared using two-tailed t-tests assuming unequal variance and donor proportion was compared using the Chi-squared test.

The sex-specific mean and standard deviation of each skeletal muscle measure were calculated independently for vertebral levels from T10 to L5. EWGSOP sarcopenia cutoffs were computed as the mean minus two standard deviations. Simultaneous differences in means for each pair of vertebral levels were tested and 95% confidence intervals generated using Tukey’s ‘Honest Significant Difference’ test^40^. Pearson correlations were used to test the linear association between measurements at each pair of vertebral levels. Agreement between sarcopenia classification for each variable and vertebra level was measured using overall accuracy, sensitivity, and specificity treating the L3 cutoffs as the reference standard.

An alpha level of 0.01 was used to determine statistical significance. All statistical tests were performed in R version 3.4.2^41^, using the package ‘ggplot2’^42^ for data visualization.

### Data availability

The datasets generated during and/or analyzed during the current study are available from the corresponding author upon reasonable request.

### Ethical Approval and Informed Consent

This study was approved by the Institutional Review Board of the University of Michigan. All methods were performed in accordance with the relevant guidelines and regulations of the United States. Because existing CT scans were used retrospectively, the requirement for informed consent was waived.

## Electronic supplementary material

Supplementary Tables
