# Supplementary material for: Skeletal muscle cutoff values for sarcopenia diagnosis using T10 to L5 measurements in a healthy US population
Source: Sci Rep. 2018 Jul 27;8:11369. doi: 10.1038/s41598-018-29825-5 (PMC6063941; doi:10.1038/s41598-018-29825-5)
Supplement: Supplementary file 1 — Supplementary Tables [file 41598_2018_29825_MOESM1_ESM.docx]

**Skeletal muscle cutoff values for sarcopenia diagnosis using T10 to L5 measurements in a healthy US population.**

**Brian A. Derstine, MS**1,***, Sven A Holcombe, PhD**1**, Brian E Ross, BS**1**, Nicholas C Wang, BS**1**, Grace L Su, MD**1,2,3**, and Stewart C Wang, MD, PhD**1,4

1Morphomic Analysis Group, University of Michigan, Ann Arbor, MI, USA 2Department of Internal Medicine, University of Michigan, Ann Arbor, MI, USA 3Department of Medicine, VA Ann Arbor Healthcare System, Ann Arbor, MI, USA 4Department of Surgery, University of Michigan, Ann Arbor, MI, USA
* bderstin@med.umich.edu

**Supplemental Tables**

|  | Female | | | |  | Male | | | |
| --- | --- | --- | --- | --- | --- | --- | --- | --- | --- |
| VB pair | n | mean | 95% CI | p |  | n | mean | 95% CI | p |
| (L5,L3) | 294 | 13.02 | (-16.96,-9.09) | 0.000 |  | 211 | 19.11 | (-25.8,-12.42) | 0.000 |
| (L4,L3) | 398 | 2.47 | (-6.09,1.16) | 0.439 |  | 305 | 18.01 | (-24.04,-11.97) | 0.000 |
| (L2,L3) | 410 | 10.24 | (-13.84,-6.65) | 0.000 |  | 315 | 11.3 | (-17.29,-5.32) | 0.000 |
| (L1,L3) | 408 | 24.55 | (-28.15,-20.95) | 0.000 |  | 315 | 35.98 | (-41.96,-29.99) | 0.000 |
| (T12,L3) | 400 | 36.64 | (-40.26,-33.03) | 0.000 |  | 299 | 54 | (-60.06,-47.93) | 0.000 |
| (T11,L3) | 335 | 44.47 | (-48.26,-40.68) | 0.000 |  | 241 | 63.45 | (-69.88,-57.02) | 0.000 |
| (T10,L3) | 156 | 41.34 | (-46.19,-36.5) | 0.000 |  | 122 | 59.81 | (-67.83,-51.8) | 0.000 |
|  |  |  |  |  |  |  |  |  |  |
| (L4,L5) | 295 | 10.56 | (6.6,14.51) | 0.000 |  | 211 | 1.1 | (-5.63,7.84) | 1.000 |
| (L2,L5) | 295 | 2.78 | (-1.15,6.71) | 0.387 |  | 209 | 7.81 | (1.11,14.5) | 0.010 |
| (L1,L5) | 294 | 11.52 | (-15.46,-7.59) | 0.000 |  | 209 | 16.87 | (-23.56,-10.17) | 0.000 |
| (T12,L5) | 289 | 23.62 | (-27.58,-19.67) | 0.000 |  | 198 | 34.89 | (-41.65,-28.12) | 0.000 |
| (T11,L5) | 243 | 31.45 | (-35.56,-27.34) | 0.000 |  | 156 | 44.34 | (-51.44,-37.25) | 0.000 |
| (T10,L5) | 107 | 28.32 | (-33.42,-23.22) | 0.000 |  | 73 | 40.7 | (-49.26,-32.15) | 0.000 |
|  |  |  |  |  |  |  |  |  |  |
| (L2,L4) | 399 | 7.78 | (-11.4,-4.16) | 0.000 |  | 303 | 6.71 | (0.66,12.75) | 0.018 |
| (L1,L4) | 397 | 22.08 | (-25.71,-18.46) | 0.000 |  | 303 | 17.97 | (-24.02,-11.93) | 0.000 |
| (T12,L4) | 389 | 34.18 | (-37.82,-30.53) | 0.000 |  | 288 | 35.99 | (-42.11,-29.87) | 0.000 |
| (T11,L4) | 326 | 42.01 | (-45.82,-38.19) | 0.000 |  | 231 | 45.44 | (-51.93,-38.96) | 0.000 |
| (T10,L4) | 150 | 38.88 | (-43.74,-34.01) | 0.000 |  | 118 | 41.81 | (-49.87,-33.75) | 0.000 |
|  |  |  |  |  |  |  |  |  |  |
| (L1,L2) | 409 | 14.3 | (-17.9,-10.7) | 0.000 |  | 315 | 24.68 | (-30.67,-18.68) | 0.000 |
| (T12,L2) | 401 | 26.4 | (-30.02,-22.78) | 0.000 |  | 299 | 42.69 | (-48.77,-36.62) | 0.000 |
| (T11,L2) | 336 | 34.23 | (-38.02,-30.44) | 0.000 |  | 241 | 52.15 | (-58.59,-45.71) | 0.000 |
| (T10,L2) | 156 | 31.1 | (-35.95,-26.25) | 0.000 |  | 122 | 48.51 | (-56.54,-40.49) | 0.000 |
|  |  |  |  |  |  |  |  |  |  |
| (T12,L1) | 401 | 12.1 | (-15.72,-8.48) | 0.000 |  | 299 | 18.02 | (-24.09,-11.94) | 0.000 |
| (T11,L1) | 336 | 19.93 | (-23.72,-16.13) | 0.000 |  | 241 | 27.47 | (-33.91,-21.03) | 0.000 |
| (T10,L1) | 156 | 16.8 | (-21.65,-11.95) | 0.000 |  | 122 | 23.84 | (-31.86,-15.81) | 0.000 |
|  |  |  |  |  |  |  |  |  |  |
| (T11,T12) | 336 | 7.83 | (-11.64,-4.02) | 0.000 |  | 241 | 9.46 | (-15.97,-2.94) | 0.000 |
| (T10,T12) | 156 | 4.7 | (-9.56,0.16) | 0.067 |  | 122 | 5.82 | (-13.9,2.27) | 0.362 |
|  |  |  |  |  |  |  |  |  |  |
| (T10,T11) | 156 | 3.13 | (-1.86,8.12) | 0.550 |  | 122 | 3.64 | (-4.72,12) | 0.891 |
| **Supplemental Table S1.** SMA mean differences for each vertebral level pair | | | | | | | | | |

|  | Female | | | |  | Male | | | |
| --- | --- | --- | --- | --- | --- | --- | --- | --- | --- |
| VB pair | n | mean | 95% CI | p |  | n | mean | 95% CI | p |
| (L5,L3) | 294 | 4.72 | (-6.19,-3.26) | 0.000 |  | 211 | 6.21 | (-8.26,-4.16) | 0.000 |
| (L4,L3) | 398 | 0.87 | (-2.22,0.49) | 0.520 |  | 305 | 5.65 | (-7.5,-3.8) | 0.000 |
| (L2,L3) | 410 | 3.79 | (-5.13,-2.45) | 0.000 |  | 315 | 3.5 | (-5.34,-1.67) | 0.000 |
| (L1,L3) | 408 | 9.09 | (-10.43,-7.75) | 0.000 |  | 315 | 11.22 | (-13.05,-9.38) | 0.000 |
| (T12,L3) | 400 | 13.54 | (-14.89,-12.19) | 0.000 |  | 299 | 16.86 | (-18.72,-15) | 0.000 |
| (T11,L3) | 335 | 16.52 | (-17.93,-15.11) | 0.000 |  | 241 | 19.81 | (-21.78,-17.84) | 0.000 |
| (T10,L3) | 156 | 15.23 | (-17.03,-13.42) | 0.000 |  | 122 | 18.77 | (-21.23,-16.31) | 0.000 |
|  |  |  |  |  |  |  |  |  |  |
| (L4,L5) | 295 | 3.86 | (2.38,5.33) | 0.000 |  | 211 | 0.56 | (-1.5,2.63) | 0.992 |
| (L2,L5) | 295 | 0.93 | (-0.54,2.4) | 0.535 |  | 209 | 2.71 | (0.66,4.76) | 0.002 |
| (L1,L5) | 294 | 4.37 | (-5.83,-2.9) | 0.000 |  | 209 | 5.01 | (-7.06,-2.95) | 0.000 |
| (T12,L5) | 289 | 8.82 | (-10.29,-7.35) | 0.000 |  | 198 | 10.65 | (-12.73,-8.58) | 0.000 |
| (T11,L5) | 243 | 11.8 | (-13.33,-10.26) | 0.000 |  | 156 | 13.6 | (-15.78,-11.43) | 0.000 |
| (T10,L5) | 107 | 10.5 | (-12.4,-8.6) | 0.000 |  | 73 | 12.56 | (-15.19,-9.94) | 0.000 |
|  |  |  |  |  |  |  |  |  |  |
| (L2,L4) | 399 | 2.93 | (-4.28,-1.58) | 0.000 |  | 303 | 2.14 | (0.29,4) | 0.011 |
| (L1,L4) | 397 | 8.22 | (-9.57,-6.87) | 0.000 |  | 303 | 5.57 | (-7.42,-3.72) | 0.000 |
| (T12,L4) | 389 | 12.68 | (-14.04,-11.32) | 0.000 |  | 288 | 11.22 | (-13.09,-9.34) | 0.000 |
| (T11,L4) | 326 | 15.65 | (-17.08,-14.23) | 0.000 |  | 231 | 14.17 | (-16.15,-12.18) | 0.000 |
| (T10,L4) | 150 | 14.36 | (-16.17,-12.55) | 0.000 |  | 118 | 13.13 | (-15.6,-10.65) | 0.000 |
|  |  |  |  |  |  |  |  |  |  |
| (L1,L2) | 409 | 5.29 | (-6.64,-3.95) | 0.000 |  | 315 | 7.71 | (-9.55,-5.88) | 0.000 |
| (T12,L2) | 401 | 9.75 | (-11.1,-8.4) | 0.000 |  | 299 | 13.36 | (-15.22,-11.5) | 0.000 |
| (T11,L2) | 336 | 12.73 | (-14.14,-11.31) | 0.000 |  | 241 | 16.31 | (-18.28,-14.34) | 0.000 |
| (T10,L2) | 156 | 11.43 | (-13.24,-9.63) | 0.000 |  | 122 | 15.27 | (-17.73,-12.81) | 0.000 |
|  |  |  |  |  |  |  |  |  |  |
| (T12,L1) | 401 | 4.46 | (-5.81,-3.11) | 0.000 |  | 299 | 5.65 | (-7.51,-3.78) | 0.000 |
| (T11,L1) | 336 | 7.43 | (-8.84,-6.02) | 0.000 |  | 241 | 8.6 | (-10.57,-6.62) | 0.000 |
| (T10,L1) | 156 | 6.14 | (-7.94,-4.33) | 0.000 |  | 122 | 7.56 | (-10.02,-5.1) | 0.000 |
|  |  |  |  |  |  |  |  |  |  |
| (T11,T12) | 336 | 2.98 | (-4.4,-1.55) | 0.000 |  | 241 | 2.95 | (-4.95,-0.95) | 0.000 |
| (T10,T12) | 156 | 1.68 | (-3.49,0.13) | 0.092 |  | 122 | 1.91 | (-4.39,0.57) | 0.273 |
|  |  |  |  |  |  |  |  |  |  |
| (T10,T11) | 156 | 1.29 | (-0.57,3.15) | 0.410 |  | 122 | 1.04 | (-1.52,3.6) | 0.922 |
| **Supplemental Table S2.** SMI mean differences for each vertebral level pair | | | | | | | | | |

|  | Female | | | |  | Male | | | |
| --- | --- | --- | --- | --- | --- | --- | --- | --- | --- |
| VB pair | n | mean | 95% CI | p |  | n | mean | 95% CI | p |
| (L5,L3) | 294 | 1.02 | (-2.38,0.33) | 0.302 |  | 211 | 0.88 | (-0.56,2.31) | 0.585 |
| (L4,L3) | 398 | 1.86 | (-3.11,-0.61) | 0.000 |  | 305 | 1.37 | (-2.67,-0.07) | 0.030 |
| (L2,L3) | 410 | 1.48 | (-2.72,-0.24) | 0.007 |  | 315 | 0.91 | (-2.19,0.38) | 0.390 |
| (L1,L3) | 408 | 2.29 | (-3.53,-1.05) | 0.000 |  | 315 | 1.5 | (-2.78,-0.21) | 0.010 |
| (T12,L3) | 400 | 0.9 | (-2.15,0.35) | 0.358 |  | 299 | 0.8 | (-2.1,0.51) | 0.581 |
| (T11,L3) | 335 | 1.53 | (-2.84,-0.22) | 0.009 |  | 241 | 2.53 | (-3.91,-1.15) | 0.000 |
| (T10,L3) | 156 | 4.51 | (-6.18,-2.83) | 0.000 |  | 122 | 5.25 | (-6.97,-3.53) | 0.000 |
|  |  |  |  |  |  |  |  |  |  |
| (L4,L5) | 295 | 0.84 | (-2.2,0.52) | 0.574 |  | 211 | 2.25 | (-3.69,-0.8) | 0.000 |
| (L2,L5) | 295 | 0.46 | (-1.82,0.89) | 0.970 |  | 209 | 1.78 | (-3.22,-0.35) | 0.004 |
| (L1,L5) | 294 | 1.27 | (-2.63,0.08) | 0.085 |  | 209 | 2.37 | (-3.81,-0.94) | 0.000 |
| (T12,L5) | 289 | 0.12 | (-1.24,1.48) | 1.000 |  | 198 | 1.67 | (-3.13,-0.22) | 0.011 |
| (T11,L5) | 243 | 0.51 | (-1.93,0.91) | 0.959 |  | 156 | 3.41 | (-4.93,-1.89) | 0.000 |
| (T10,L5) | 107 | 3.48 | (-5.24,-1.73) | 0.000 |  | 73 | 6.12 | (-7.96,-4.29) | 0.000 |
|  |  |  |  |  |  |  |  |  |  |
| (L2,L4) | 399 | 0.38 | (-0.87,1.63) | 0.984 |  | 303 | 0.46 | (-0.84,1.76) | 0.961 |
| (L1,L4) | 397 | 0.43 | (-1.68,0.82) | 0.967 |  | 303 | 0.13 | (-1.43,1.17) | 1.000 |
| (T12,L4) | 389 | 0.96 | (-0.3,2.22) | 0.283 |  | 288 | 0.57 | (-0.74,1.89) | 0.892 |
| (T11,L4) | 326 | 0.33 | (-0.99,1.64) | 0.995 |  | 231 | 1.16 | (-2.56,0.23) | 0.182 |
| (T10,L4) | 150 | 2.64 | (-4.32,-0.97) | 0.000 |  | 118 | 3.88 | (-5.61,-2.15) | 0.000 |
|  |  |  |  |  |  |  |  |  |  |
| (L1,L2) | 409 | 0.81 | (-2.05,0.43) | 0.493 |  | 315 | 0.59 | (-1.88,0.7) | 0.862 |
| (T12,L2) | 401 | 0.58 | (-0.66,1.83) | 0.850 |  | 299 | 0.11 | (-1.2,1.41) | 1.000 |
| (T11,L2) | 336 | 0.05 | (-1.35,1.26) | 1.000 |  | 241 | 1.63 | (-3.01,-0.24) | 0.009 |
| (T10,L2) | 156 | 3.02 | (-4.69,-1.35) | 0.000 |  | 122 | 4.34 | (-6.07,-2.62) | 0.000 |
|  |  |  |  |  |  |  |  |  |  |
| (T12,L1) | 401 | 1.39 | (0.15,2.64) | 0.016 |  | 299 | 0.7 | (-0.61,2) | 0.734 |
| (T11,L1) | 336 | 0.76 | (-0.55,2.07) | 0.642 |  | 241 | 1.04 | (-2.42,0.35) | 0.310 |
| (T10,L1) | 156 | 2.21 | (-3.88,-0.54) | 0.002 |  | 122 | 3.75 | (-5.47,-2.03) | 0.000 |
|  |  |  |  |  |  |  |  |  |  |
| (T11,T12) | 336 | 0.63 | (-1.94,0.68) | 0.830 |  | 241 | 1.74 | (-3.13,-0.34) | 0.004 |
| (T10,T12) | 156 | 3.6 | (-5.28,-1.93) | 0.000 |  | 122 | 4.45 | (-6.19,-2.71) | 0.000 |
|  |  |  |  |  |  |  |  |  |  |
| (T10,T11) | 156 | 2.97 | (-4.69,-1.25) | 0.000 |  | 122 | 2.72 | (-4.51,-0.92) | 0.000 |
| **Supplemental Table S3.** SMRA mean differences for each vertebral level pair | | | | | | | | | |

|  | Female | | | |  | Male | | | |
| --- | --- | --- | --- | --- | --- | --- | --- | --- | --- |
| VB pair | n | r | 95% CI | p |  | n | r | 95% CI | p |
| (L5,L3) | 294 | 0.85 | (0.82,0.88) | <0.001 |  | 211 | 0.84 | (0.79,0.88) | <0.001 |
| (L4,L3) | 398 | 0.88 | (0.86,0.9) | <0.001 |  | 305 | 0.88 | (0.85,0.9) | <0.001 |
| (L2,L3) | 410 | 0.94 | (0.93,0.95) | <0.001 |  | 315 | 0.92 | (0.9,0.94) | <0.001 |
| (L1,L3) | 408 | 0.91 | (0.89,0.92) | <0.001 |  | 315 | 0.87 | (0.84,0.89) | <0.001 |
| (T12,L3) | 400 | 0.85 | (0.82,0.87) | <0.001 |  | 299 | 0.8 | (0.76,0.84) | <0.001 |
| (T11,L3) | 335 | 0.83 | (0.79,0.86) | <0.001 |  | 241 | 0.78 | (0.72,0.82) | <0.001 |
| (T10,L3) | 156 | 0.84 | (0.79,0.88) | <0.001 |  | 122 | 0.75 | (0.66,0.82) | <0.001 |
|  |  |  |  |  |  |  |  |  |  |
| (L4,L5) | 295 | 0.79 | (0.74,0.83) | <0.001 |  | 211 | 0.72 | (0.64,0.78) | <0.001 |
| (L2,L5) | 295 | 0.83 | (0.79,0.86) | <0.001 |  | 209 | 0.87 | (0.83,0.9) | <0.001 |
| (L1,L5) | 294 | 0.79 | (0.74,0.83) | <0.001 |  | 209 | 0.82 | (0.78,0.86) | <0.001 |
| (T12,L5) | 289 | 0.76 | (0.71,0.81) | <0.001 |  | 198 | 0.82 | (0.76,0.86) | <0.001 |
| (T11,L5) | 243 | 0.75 | (0.68,0.8) | <0.001 |  | 156 | 0.76 | (0.69,0.82) | <0.001 |
| (T10,L5) | 107 | 0.72 | (0.62,0.8) | <0.001 |  | 73 | 0.79 | (0.68,0.86) | <0.001 |
|  |  |  |  |  |  |  |  |  |  |
| (L2,L4) | 399 | 0.8 | (0.77,0.84) | <0.001 |  | 303 | 0.76 | (0.71,0.8) | <0.001 |
| (L1,L4) | 397 | 0.78 | (0.73,0.81) | <0.001 |  | 303 | 0.7 | (0.64,0.75) | <0.001 |
| (T12,L4) | 389 | 0.74 | (0.69,0.78) | <0.001 |  | 288 | 0.65 | (0.58,0.71) | <0.001 |
| (T11,L4) | 326 | 0.76 | (0.71,0.8) | <0.001 |  | 231 | 0.68 | (0.6,0.74) | <0.001 |
| (T10,L4) | 150 | 0.79 | (0.72,0.84) | <0.001 |  | 118 | 0.69 | (0.58,0.77) | <0.001 |
|  |  |  |  |  |  |  |  |  |  |
| (L1,L2) | 409 | 0.95 | (0.93,0.96) | <0.001 |  | 315 | 0.95 | (0.94,0.96) | <0.001 |
| (T12,L2) | 401 | 0.9 | (0.88,0.92) | <0.001 |  | 299 | 0.89 | (0.87,0.91) | <0.001 |
| (T11,L2) | 336 | 0.86 | (0.84,0.89) | <0.001 |  | 241 | 0.83 | (0.79,0.87) | <0.001 |
| (T10,L2) | 156 | 0.86 | (0.81,0.9) | <0.001 |  | 122 | 0.78 | (0.7,0.84) | <0.001 |
|  |  |  |  |  |  |  |  |  |  |
| (T12,L1) | 401 | 0.93 | (0.92,0.94) | <0.001 |  | 299 | 0.92 | (0.91,0.94) | <0.001 |
| (T11,L1) | 336 | 0.89 | (0.87,0.91) | <0.001 |  | 241 | 0.86 | (0.82,0.89) | <0.001 |
| (T10,L1) | 156 | 0.87 | (0.83,0.91) | <0.001 |  | 122 | 0.81 | (0.74,0.86) | <0.001 |
|  |  |  |  |  |  |  |  |  |  |
| (T11,T12) | 336 | 0.92 | (0.91,0.94) | <0.001 |  | 241 | 0.91 | (0.88,0.93) | <0.001 |
| (T10,T12) | 156 | 0.89 | (0.85,0.92) | <0.001 |  | 122 | 0.83 | (0.77,0.88) | <0.001 |
|  |  |  |  |  |  |  |  |  |  |
| (T10,T11) | 156 | 0.93 | (0.9,0.95) | <0.001 |  | 122 | 0.87 | (0.82,0.91) | <0.001 |
| **Supplemental Table S4.** SMA Pearson correlation for each vertebral level pair | | | | | | | | | |

|  | Female | | | |  | Male | | | |
| --- | --- | --- | --- | --- | --- | --- | --- | --- | --- |
| VB pair | n | r | 95% CI | p |  | n | r | 95% CI | p |
| (L5,L3) | 294 | 0.84 | (0.81,0.87) | <0.001 |  | 211 | 0.82 | (0.77,0.86) | <0.001 |
| (L4,L3) | 398 | 0.88 | (0.86,0.9) | <0.001 |  | 305 | 0.87 | (0.84,0.89) | <0.001 |
| (L2,L3) | 410 | 0.94 | (0.93,0.95) | <0.001 |  | 315 | 0.92 | (0.91,0.94) | <0.001 |
| (L1,L3) | 408 | 0.91 | (0.89,0.93) | <0.001 |  | 315 | 0.87 | (0.84,0.89) | <0.001 |
| (T12,L3) | 400 | 0.85 | (0.82,0.87) | <0.001 |  | 299 | 0.8 | (0.76,0.84) | <0.001 |
| (T11,L3) | 335 | 0.84 | (0.81,0.87) | <0.001 |  | 241 | 0.78 | (0.72,0.82) | <0.001 |
| (T10,L3) | 156 | 0.84 | (0.79,0.88) | <0.001 |  | 122 | 0.75 | (0.66,0.82) | <0.001 |
|  |  |  |  |  |  |  |  |  |  |
| (L4,L5) | 295 | 0.78 | (0.73,0.82) | <0.001 |  | 211 | 0.69 | (0.61,0.75) | <0.001 |
| (L2,L5) | 295 | 0.82 | (0.78,0.85) | <0.001 |  | 209 | 0.86 | (0.81,0.89) | <0.001 |
| (L1,L5) | 294 | 0.78 | (0.73,0.82) | <0.001 |  | 209 | 0.8 | (0.75,0.85) | <0.001 |
| (T12,L5) | 289 | 0.75 | (0.7,0.8) | <0.001 |  | 198 | 0.8 | (0.74,0.84) | <0.001 |
| (T11,L5) | 243 | 0.75 | (0.69,0.8) | <0.001 |  | 156 | 0.74 | (0.66,0.8) | <0.001 |
| (T10,L5) | 107 | 0.67 | (0.56,0.77) | <0.001 |  | 73 | 0.75 | (0.63,0.84) | <0.001 |
|  |  |  |  |  |  |  |  |  |  |
| (L2,L4) | 399 | 0.81 | (0.77,0.84) | <0.001 |  | 303 | 0.75 | (0.7,0.8) | <0.001 |
| (L1,L4) | 397 | 0.78 | (0.74,0.82) | <0.001 |  | 303 | 0.69 | (0.62,0.74) | <0.001 |
| (T12,L4) | 389 | 0.74 | (0.69,0.78) | <0.001 |  | 288 | 0.64 | (0.57,0.71) | <0.001 |
| (T11,L4) | 326 | 0.78 | (0.73,0.82) | <0.001 |  | 231 | 0.66 | (0.58,0.73) | <0.001 |
| (T10,L4) | 150 | 0.79 | (0.72,0.84) | <0.001 |  | 118 | 0.65 | (0.53,0.75) | <0.001 |
|  |  |  |  |  |  |  |  |  |  |
| (L1,L2) | 409 | 0.95 | (0.93,0.96) | <0.001 |  | 315 | 0.95 | (0.94,0.96) | <0.001 |
| (T12,L2) | 401 | 0.91 | (0.89,0.92) | <0.001 |  | 299 | 0.89 | (0.87,0.91) | <0.001 |
| (T11,L2) | 336 | 0.88 | (0.85,0.9) | <0.001 |  | 241 | 0.83 | (0.79,0.87) | <0.001 |
| (T10,L2) | 156 | 0.86 | (0.81,0.89) | <0.001 |  | 122 | 0.78 | (0.7,0.84) | <0.001 |
|  |  |  |  |  |  |  |  |  |  |
| (T12,L1) | 401 | 0.93 | (0.92,0.94) | <0.001 |  | 299 | 0.92 | (0.91,0.94) | <0.001 |
| (T11,L1) | 336 | 0.9 | (0.88,0.92) | <0.001 |  | 241 | 0.86 | (0.82,0.89) | <0.001 |
| (T10,L1) | 156 | 0.87 | (0.83,0.91) | <0.001 |  | 122 | 0.8 | (0.73,0.86) | <0.001 |
|  |  |  |  |  |  |  |  |  |  |
| (T11,T12) | 336 | 0.93 | (0.91,0.94) | <0.001 |  | 241 | 0.91 | (0.88,0.93) | <0.001 |
| (T10,T12) | 156 | 0.89 | (0.85,0.92) | <0.001 |  | 122 | 0.82 | (0.76,0.87) | <0.001 |
|  |  |  |  |  |  |  |  |  |  |
| (T10,T11) | 156 | 0.93 | (0.9,0.95) | <0.001 |  | 122 | 0.86 | (0.81,0.9) | <0.001 |
| **Supplemental Table S5.** SMI Pearson correlation for each vertebral level pair | | | | | | | | | |

|  | Female | | | |  | Male | | | |
| --- | --- | --- | --- | --- | --- | --- | --- | --- | --- |
| VB pair | n | r | 95% CI | p |  | n | r | 95% CI | p |
| (L5,L3) | 294 | 0.87 | (0.84,0.9) | <0.001 |  | 211 | 0.93 | (0.91,0.95) | <0.001 |
| (L4,L3) | 398 | 0.92 | (0.91,0.94) | <0.001 |  | 305 | 0.93 | (0.92,0.95) | <0.001 |
| (L2,L3) | 410 | 0.94 | (0.93,0.95) | <0.001 |  | 315 | 0.95 | (0.94,0.96) | <0.001 |
| (L1,L3) | 408 | 0.88 | (0.85,0.9) | <0.001 |  | 315 | 0.92 | (0.9,0.93) | <0.001 |
| (T12,L3) | 400 | 0.85 | (0.81,0.87) | <0.001 |  | 299 | 0.85 | (0.82,0.88) | <0.001 |
| (T11,L3) | 335 | 0.78 | (0.73,0.82) | <0.001 |  | 241 | 0.74 | (0.68,0.79) | <0.001 |
| (T10,L3) | 156 | 0.73 | (0.65,0.8) | <0.001 |  | 122 | 0.78 | (0.7,0.84) | <0.001 |
|  |  |  |  |  |  |  |  |  |  |
| (L4,L5) | 295 | 0.83 | (0.79,0.86) | <0.001 |  | 211 | 0.9 | (0.88,0.93) | <0.001 |
| (L2,L5) | 295 | 0.84 | (0.81,0.87) | <0.001 |  | 209 | 0.91 | (0.88,0.93) | <0.001 |
| (L1,L5) | 294 | 0.76 | (0.71,0.81) | <0.001 |  | 209 | 0.88 | (0.84,0.91) | <0.001 |
| (T12,L5) | 289 | 0.73 | (0.67,0.78) | <0.001 |  | 198 | 0.84 | (0.79,0.87) | <0.001 |
| (T11,L5) | 243 | 0.68 | (0.61,0.74) | <0.001 |  | 156 | 0.71 | (0.62,0.78) | <0.001 |
| (T10,L5) | 107 | 0.63 | (0.5,0.73) | <0.001 |  | 73 | 0.77 | (0.65,0.85) | <0.001 |
|  |  |  |  |  |  |  |  |  |  |
| (L2,L4) | 399 | 0.89 | (0.87,0.91) | <0.001 |  | 303 | 0.91 | (0.89,0.93) | <0.001 |
| (L1,L4) | 397 | 0.83 | (0.8,0.86) | <0.001 |  | 303 | 0.9 | (0.87,0.92) | <0.001 |
| (T12,L4) | 389 | 0.82 | (0.78,0.85) | <0.001 |  | 288 | 0.85 | (0.82,0.88) | <0.001 |
| (T11,L4) | 326 | 0.75 | (0.69,0.79) | <0.001 |  | 231 | 0.72 | (0.65,0.78) | <0.001 |
| (T10,L4) | 150 | 0.73 | (0.65,0.8) | <0.001 |  | 118 | 0.78 | (0.69,0.84) | <0.001 |
|  |  |  |  |  |  |  |  |  |  |
| (L1,L2) | 409 | 0.86 | (0.84,0.89) | <0.001 |  | 315 | 0.91 | (0.89,0.93) | <0.001 |
| (T12,L2) | 401 | 0.83 | (0.8,0.86) | <0.001 |  | 299 | 0.86 | (0.82,0.88) | <0.001 |
| (T11,L2) | 336 | 0.74 | (0.69,0.79) | <0.001 |  | 241 | 0.74 | (0.68,0.79) | <0.001 |
| (T10,L2) | 156 | 0.71 | (0.62,0.78) | <0.001 |  | 122 | 0.79 | (0.72,0.85) | <0.001 |
|  |  |  |  |  |  |  |  |  |  |
| (T12,L1) | 401 | 0.9 | (0.87,0.91) | <0.001 |  | 299 | 0.88 | (0.85,0.9) | <0.001 |
| (T11,L1) | 336 | 0.76 | (0.72,0.81) | <0.001 |  | 241 | 0.76 | (0.7,0.81) | <0.001 |
| (T10,L1) | 156 | 0.72 | (0.64,0.79) | <0.001 |  | 122 | 0.78 | (0.7,0.84) | <0.001 |
|  |  |  |  |  |  |  |  |  |  |
| (T11,T12) | 336 | 0.86 | (0.83,0.89) | <0.001 |  | 241 | 0.83 | (0.79,0.87) | <0.001 |
| (T10,T12) | 156 | 0.77 | (0.7,0.83) | <0.001 |  | 122 | 0.79 | (0.71,0.85) | <0.001 |
|  |  |  |  |  |  |  |  |  |  |
| (T10,T11) | 156 | 0.89 | (0.85,0.92) | <0.001 |  | 122 | 0.88 | (0.83,0.91) | <0.001 |
| **Supplemental Table S6.** SMRA Pearson correlation for each vertebral level pair | | | | | | | | | |
